# Activity-driven network modeling and control of the spread of two concurrent epidemic strains

**DOI:** 10.1007/s41109-022-00507-6

**Published:** 2022-09-27

**Authors:** Daniel Alberto Burbano Lombana, Lorenzo Zino, Sachit Butail, Emanuele Caroppo, Zhong-Ping Jiang, Alessandro Rizzo, Maurizio Porfiri

**Affiliations:** 1 Center for Urban Science and Progress, Tandon School of Engineering, New York University, 370 Jay Street, Brooklyn, NY 11201 USA; 2 Department of Mechanical and Aerospace Engineering, Tandon School of Engineering, New York University, Six MetroTech Center, Brooklyn, NY 11201 USA; 3 Engineering and Technology Institute Groningen, University of Groningen, Nijenborgh 4, 9747 AG Groningen, The Netherlands; 4 Department of Mechanical Engineering, Northern Illinois University, DeKalb, IL 60115 USA; 5 Department of Mental Health, Local Health Unit Roma 2, 00159 Rome, Italy; 6 University Research Center He.R.A., Universitá Cattolica del Sacro Cuore, 00168 Rome, Italy; 7 Department of Electrical and Computer Engineering, Tandon School of Engineering, New York University, 370 Jay Street, Brooklyn, NY 11201 USA; 8 Department of Electronics and Telecommunications, Politecnico di Torino, Corso Duca Degli Abruzzi, 24, 10129 Turin, Italy; 9 Institute for Invention, Innovation and Entrepreneurship, Tandon School of Engineering, New York University, Six MetroTech Center, Brooklyn, NY 11201 USA; 10 Department of Biomedical Engineering, Tandon School of Engineering, New York University, Six MetroTech Center, Brooklyn, NY 11201 USA; 11 Department of Electrical and Computer Engineering, Rutgers University, 94 Brett Rd, Piscataway, NJ 08854 USA

**Keywords:** Bi-virus, Complex networks, Control, Epidemics, Temporal network

## Abstract

The emergency generated by the current COVID-19 pandemic has claimed millions of lives worldwide. There have been multiple waves across the globe that emerged as a result of new variants, due to arising from unavoidable mutations. The existing network toolbox to study epidemic spreading cannot be readily adapted to the study of multiple, coexisting strains. In this context, particularly lacking are models that could elucidate re-infection with the same strain or a different strain—phenomena that we are seeing experiencing more and more with COVID-19. Here, we establish a novel mathematical model to study the simultaneous spreading of two strains over a class of temporal networks. We build on the classical susceptible–exposed–infectious–removed model, by incorporating additional states that account for infections and re-infections with multiple strains. The temporal network is based on the activity-driven network paradigm, which has emerged as a model of choice to study dynamic processes that unfold at a time scale comparable to the network evolution. We draw analytical insight from the dynamics of the stochastic network systems through a mean-field approach, which allows for characterizing the onset of different behavioral phenotypes (non-epidemic, epidemic, and endemic). To demonstrate the practical use of the model, we examine an intermittent stay-at-home containment strategy, in which a fraction of the population is randomly required to isolate for a fixed period of time.

## Introduction

During the spread of an infectious disease, viral mutations may weaken public health measures as new transmission dynamics emerge that lessen the effects of vaccination and cause unseen comorbidities. For instance, influenza exhibits a high mutation rate in the viral genome that can evolve into new virus strains (Andreasen et al. 1997). In addition, empirical evidence of monkeypox indicates that a single mutation may produce genetic variation that can lead to the emergence of a new variant (Douglass et al. 1994). In the ongoing COVID-19 pandemic, we have been experiencing a similar scenario, with several SARS-CoV-2 variants propagating across the globe (Duong 2021). As of July 2022, we are currently witnessing several *Omicron* sub-variants, such as the BA.1 that emerged at the end of 2021 in Botswana and South Africa (Phan et al. 2022) and the BA.5 that is threatening vaccine-induced immunity in the USA (Callaway 2021; Grubaugh and Cobey 2021).

Mathematical models of infectious diseases offer important insights into the spreading process of diseases, transmitted by interactions between individuals while providing a framework to devise containment strategies. The literature on mathematical modeling of disease spreading has proliferated during the COVID-19 pandemic and several approaches have been developed at different levels of resolution (Brauer 2017; Bertozzi et al. 2020). Low-resolution models typically partition the population into a finite number of compartments and describe their rate of change through a set of differential equations. While these models may have limited predictive value, they allow for a simple mathematical treatment that can shed light on the macroscopic epidemic behavior and highlight the role and criticality of model parameters.

Low-resolution models have been recently proposed to study the effect of multiple strains. For instance, in Fudolig and Howard (2020), an extension of the classical susceptible–infected–removed (SIR) model with mutations, re-infection, and compartments accounting for vaccinated individuals has been proposed to model the spread of a virus with a nominal strain and an emergent one that is vaccine-resistant. The authors examined the local stability of four different equilibria, corresponding to the case in which both variants vanish, the cases in which one variant vanishes and the other persists, and the case in which both variants persist over time. In de León et al. (2022), the authors considered additional states, such as infected-but-asymptomatic and dead, to model the spread of COVID-19 with two variants. In Arruda et al. (2021), the authors proposed a multi-strain epidemic model, along with an optimal control approach to contain the spread.

At the other end of the spectrum, agent-based models (ABMs) can reproduce the behavior of a population with great granularity (Azizi et al. 2021; Aleta et al. 2020; Truszkowska et al. 2021; Kerr et al. 2021). For instance, in Azizi et al. (2021), the authors developed an ABM based on the SIR dynamics to investigate the role of human behavior, in the form of self-regulated or mandated social distancing, on the spread of a virus with two strains. Likewise, in Truszkowska et al. (2021), an ABM at the resolution of a single individual was created to study the propagation of COVID-19 in a real town in the United States. A theoretical analysis of these high-resolution models is difficult, if not impossible, due to the complexity of the dynamics, the stochasticity of the spreading, and the large parameter space.

Network theory constitutes a modeling pathway at an intermediate resolution, which allows for some analytical treatment in the spirit of compartmental models, while granting some fineness in the description of spreading like ABMs (Paré et al. 2020; Zino and Cao 2021; Pastor-Satorras et al. 2015; Mei et al. 2017; Kiss et al. 2017). Through the lens of networks, individuals are modeled as the nodes of a graph who interact through the edges of the network of contacts. Such a network captures the interactions between individuals, through which most viral diseases spread, such as contact with infected body fluids (Grant et al. 2020; Azmat et al. 2021), respiratory droplets, or aerosol generated when a person coughs, sneezes, or simply speaks (Killingley and Nguyen-Van-Tam 2013; Jayaweera et al. 2020; Netz and Eaton 2020).

Within the context of network epidemic models, some efforts have been made to study the spread of multiple viruses and variants. In Bansal and Meyers (2012), the authors developed a model to study consecutive outbreaks with partial immunity after recovery, using percolation theory. In Karrer and Newman (2011), a model of two concurrent diseases spreading over the same static networks of contacts was established, detailing the transition between the dominance of each disease over the other and the presence of a regime in which both co-exist. In Prakash et al. (2012), it was shown that co-existence is a rare phenomenon in most real-world network structures, where one disease typically dominates the other. A similar study on metapopulation model helped clarify the role of the network structure on the transitions between different regimes (Poletto et al. 2013). This modeling framework was extended to account for diseases concurrently spreading on distinct networks of contacts (Sanz et al. 2014) or on multi-layer networks (Sahneh and Scoglio 2014). It has been shown that the network model paradigm can be utilized to study real-world scenarios (Pinotti et al. 2019) while allowing to establish rigorous analytical treatment, towards the designing techniques to contain the viral spread (Liu et al. 2019; Paré et al. 2021; Ye et al. 2022).

While early accounts considered static networks (Mei et al. 2017; Fall et al. 2007), there is a general consensus that temporal networks should be preferred to capture the dynamic nature of human behavior and interactions (Zino and Cao 2021; Pastor-Satorras et al. 2015; Prakash et al. 2010). Activity-driven networks (ADNs) (Perra et al. 2012) have emerged as an elegant framework to study spreading dynamics over temporal networks in which the network dynamics evolve at the same time scale of the unfolding disease spreading. This modeling approach allows for mathematical treatment and provides important insights on how the node and network dynamics both contribute to the overall spreading process (Perra et al. 2012; Liu et al. 2014; Rizzo et al. 2014, 2016; Zino et al. 2016; Lei et al. 2016; Pozzana et al. 2017; Ogura et al. 2019; Behring et al. 2021).

Here, we extend the ADN paradigm to study the simultaneous propagation of two strains, building on the literature on bi-virus susceptible–infected–susceptible (SIS) models (Prakash et al. 2012; Sahneh and Scoglio 2014; Liu et al. 2019; Paré et al. 2021; Ye et al. 2022). In an effort to tackle realistic disease spreading, from COVID-19 to influenza, dengue, and malaria (Kucharski et al. 2016), we formulate the problem within a susceptible–exposed–infected–removed (SEIR) model and consider re-infections with tunable parameters for virus-specific and cross immunity. Our modeling framework captures a rich behavioral repertoire where both strains can spread simultaneously or independently, also contemplating the scenario of an endemic state with coexisting variants. Specifically, we characterize three different types of behavior: (i) quick eradication of the disease, (ii) eradication of the disease after the occurrence of an epidemic outbreak, and (iii) emergence of an endemic disease. Through a mean-field approach (Van Mieghem et al. 2009; Perra et al. 2012), we establish simple algebraic conditions determining the stability of the disease-free equilibrium and endemic states.

To demonstrate the practical value of our modeling approach, we propose the implementation of a non-pharmaceutical intervention, in the form of an intermittent stay-at-home strategy. Non-pharmaceutical interventions are key to limit transmission routes between individuals (Markel et al. 2007; Flaxman et al. 2020; Di Domenico et al. 2020; Arenas et al. 2020; Perra 2021) before vaccines become available for mass use. In particular, intermittent strategies have been examined in Valdez et al. (2012), where the authors have studied the role of intermittent social distancing in a static network model with SIS dynamics. In this vein, individuals might interrupt interactions with those infected for a fixed period of time to then resume contact. In Meidan et al. (2021), a similar control strategy has been studied for potential implementation in the fight against COVID-19. Similarly, in Della Rossa et al. (2020), the authors have examined how an intermittent strategy at a regional level in Italy can mitigate the effects of the COVID-19 spread, and an equivalent analysis has been carried out in Bin et al. (2021) for fast-switching control.

The rest of the paper is organized as follows. In the “Model” section, we present the model and provide an example illustrating its rich dynamic repertoire. In the “Mean-field analysis” section, we conduct a mean-field analysis to predict the regions in the parameter space where the system either converges to the disease-free equilibrium or the endemic state. We present the intermittent stay-at-home control strategy in the “Intermittent stay-at-home containment strategy” section, while conclusions and future work are presented in the “Conclusions” section.

## Model

We consider a set of *N* nodes, each associated with an individual, which interact through a temporal network represented by an undirected graph ${\mathcal {G}}(t) = ({\mathcal {N}} , {\mathcal {E}}(t))$, where ${\mathcal {N}}:= \{1,\ldots , N \}$ is the node set and ${\mathcal {E}}(t)\subseteq {\mathcal {N}}\times {\mathcal {N}}$ is the edge set—$(i,j)\in {\mathcal {E}}(t)$ means that individuals *i* and *j* are in contact at time *t*. Here, *t* denotes the discrete time variable $t\in \{0,\Delta ,2\Delta ,3\Delta ,\ldots \}$, with $\Delta >0$ being the time step.

Consistent with the literature on bi- and multi-virus models (Prakash et al. 2012; Sahneh and Scoglio 2014; Liu et al. 2019; Paré et al. 2021; Ye et al. 2022), we assume that individuals can be exposed to or be infected with at most one of two different strains of the virus at the same time. As such, an individual cannot carry both strains at the same time. Upon recovery from an infection, individuals gain (partial) strain-specific (Stokel-Walker 2021; Iwasaki 2021; Ren et al. 2022) and cross-strain immunity, so that they can still be re-infected, but with a reduced probability (Andreasen et al. 1997; Kaler et al. 2022).

### Node dynamics

**Fig. 1:**
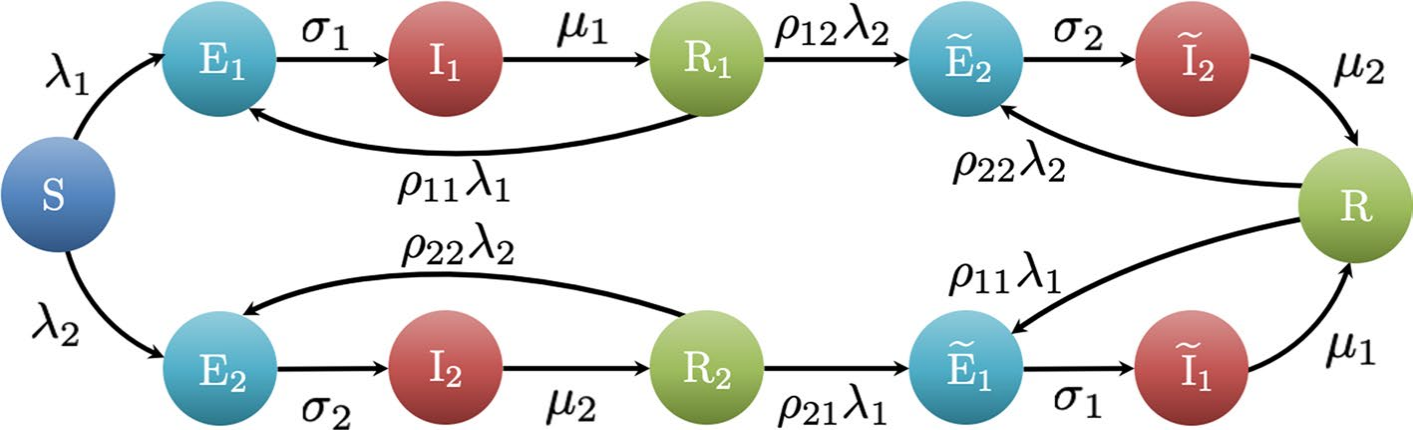
Progression of a virus spread with two strains. The diagram describes the transitions that each individual undergoes between health states. All parameters are constant and represent transition probabilities or rates

Taking into account these considerations, for each individual (represented by a node in the network) we consider the progression illustrated in Fig. 1—a bi-virus version of an SEIR model. The health state of each individual, denoted by $x_i(t)\in {\mathcal {X}}$ for all $i\in {\mathcal {N}}$, can take values in ${\mathcal {X}}:=\{\text {S},\text {E}_1,\text {E}_2,\text {I}_1,\text {I}_2,\text {R}_1,\text {R}_2,\widetilde{\text {E}}_1,\widetilde{\text {E}}_2,\widetilde{\text {I}}_1,\widetilde{\text {I}}_2,\text {R}\}$. Here, $\text {S}$ denotes the susceptible state, in which the individual is healthy and can potentially become infected, as they come in contact with infectious individuals.

Upon infection, the health state of an individual changes to exposed, denoted by $\text {E}_\ell$, where the index $\ell \in \{1,2\}$ refers to the strain the individual has been exposed to. In this state, the virus within an individual is in an incubation phase, so that the individual is infected, but cannot transmit the disease yet. The incubation phase lasts for a stochastic time interval. Specifically, at each time step, an individual who is exposed to strain $\ell \in \{1,2\}$ transitions to the infectious state ($\text {I}_\ell$) with probability (w.p.) $\sigma _\ell \Delta$, independent of the other individuals and of the past. Infected individuals can transmit the disease with a duration of the infection also governed by a stochastic mechanism: at each time step, an individual who is infected with strain $\ell \in \{1,2\}$ transitions to the recovered state $\text {R}_\ell$ w.p. $\mu _\ell \Delta$, independent of the others and of the past.

After recovery, an individual acquires partial immunity, so that recovered individuals can still be infected by either of the two strains, albeit with reduced probabilities compared to an individual in a susceptible state. We introduce two further pairs of progression states, marked with a tilde to model partial immunity to a strain with which an individual has been previously infected. If an individual in state $\text {R}_\ell$ is re-infected with the same strain $\ell$, they transition back to the same progression sequence; alternatively, they may be exposed to the other strain. This state is denoted by $\widetilde{\text {E}}_{\bar{\ell }}$, introduced to keep track of the partial immunity previously gained through infection; here and in what follows, we use a superimposed bar to identify the virus strain other than $\ell$. An individual who underwent an infection with both strains gains immunity against both of them, and transitions to the recovered state $\mathrm {R}$.

The contagion mechanism acts as follows. At each time step *t*, a susceptible individual ($\text {S}$) who has an interaction with an infected individual with strain $\ell \in \{1,2\}$ (${\text {I}}_\ell$ or $\widetilde{\text {I}}_\ell$) becomes exposed with per-contact infection probability equal to $\lambda _\ell$, independent of other contacts that the susceptible individual might have had. We assume that recovery from strain $\ell \in \{1,2\}$ ($\text {R}_\ell$) yields a partial strain-specific immunity against that strain and cross-strain immunity against the other strain $\bar{\ell }$. The levels of immunity are captured by the strain-specific re-infection probability $\rho _{\ell \ell }\in [0,1]$ and the cross-strain re-infection probability $\rho _{\ell \bar{\ell }}\in [0,1]$, respectively. In particular, $\rho _{\ell \ell }=1$ means that no immunity is present, while $\rho _{\ell \ell }=0$ models the ideal scenario of perfect immunity. Using these parameters, for individuals who have recovered from strain $\ell \in \{1,2\}$ (${\text {R}}_\ell$), the per-contact infection probabilities with strain $\ell$ and $\bar{\ell }$ are scaled to $\rho _{\ell \ell }\lambda _{\ell }$ and $\rho _{\ell \bar{\ell }}\lambda _{\bar{\ell }}$, respectively. Typically, strain-specific immunity is stronger than cross-strain immunity, so that we assume $\rho _{\ell \ell }\le \rho _{\bar{\ell }\ell }$. Hence, for individuals who have recovered from both strains ($\text {R}$), we scale the infection probabilities using the strain-specific re-infection probability $\rho _{\ell \ell }$ for both strains $\ell \in \{1,2\}$.

The mechanisms described above establish that the dynamics of individual $i\in {\mathcal {N}}$, with state $x_i(t+\Delta )\in \mathcal X$, are described by a Markov chain (Levin et al. 2017), with the following non-zero transition probabilities. With respect to transitions that do not involve interactions, we have

1 $$\begin{aligned} x_i(t+\Delta )&=\left\{ \begin{array}{ll} {\text {I}_\ell ,} &{} {\text {w.p. } \sigma _{\ell }\Delta ,} {\text { if } x_i(t)=\text {E}_\ell },\\ {\text {R}_\ell ,} &{} {\text {w.p. } \mu _{\ell }\Delta ,} {\text { if } x_i(t)=\text {I}_\ell },\\ {\widetilde{\text {I}}_\ell ,} &{} {\text {w.p. } \sigma _{\ell }\Delta ,} {\text { if } x_i(t)=\widetilde{\text {E}}_\ell },\\ {\text {R},} &{} {\text {w.p. } \mu _{\ell }\Delta ,} {\text { if } x_i(t)=\widetilde{\text {I}}_\ell }, \end{array}\right. \end{aligned}$$

for $\ell \in \{1,2\}$. Transitions from $\text {S}$ to $\text {E}_1$ and $\text {E}_2$ depend on interactions with neighboring individuals in the network of contacts ${\mathcal {G}}(t)$, that is,

2 $$\begin{aligned} x_i(t+\Delta ) = \text {E}_\ell , {\text { w.p. } P_\ell (i,t,1),} {\text { if } x_i(t)=\text {S}}, \end{aligned}$$

for $\ell \in \{1,2\}$. Here, the contagion probability for individual *i* at time *t* is defined as

3 $$\begin{aligned} P_\ell (i,t,r):=1-(1-r\lambda _{\ell })^{{\mathcal {I}}_\ell (i,t)}\,, \end{aligned}$$

where

4 $$\begin{aligned} {\mathcal {I}}_\ell (i,t):= {|\{j\in {\mathcal {N}}:(i,j)\in \mathcal E(t)\text { and }x_j(t)\in \{\text {I}_\ell ,\widetilde{\text {I}}_\ell \}\}|} \end{aligned}$$

is the number of neighbors of *i* at time *t* who are infectious with strain $\ell$, and $r\in [0,1]$ is an auxiliary parameter that re-scales the per-contact infection probability to account for the possible presence of a level of immunity due to previous infections. In (2), such a parameter is set to $r=1$, since susceptible individuals have no partial immunity. In plain words, equation (3) indicates that each neighbor of *i* who is infected with strain $\ell$ has a probability ${r}\lambda _\ell$ of transmitting the disease to *i*, independent of others.

Finally, transitions due to re-infection from the recovered states $\mathrm {R}_1$, $\mathrm {R}_2$, and $\mathrm {R}$ to the exposed states $\text {E}_1$, $\text {E}_2$, $\widetilde{\text {E}}_1$, and $\widetilde{\text {E}}_2$ follow a similar mechanism, with the re-scaling factor *r* in equation (3) that takes value equal to the corresponding re-infection probability. Specifically, we have

5 $$\begin{aligned} x_i(t+\Delta ) =\left\{ \begin{array}{ll} \text {E}_\ell , &{} \text {w.p. }P_\ell (i,t,\rho _{\ell \ell }), {\text { if }} x_i(t)=\text {R}_\ell ,\\ \widetilde{\text {E}}_\ell , &{} {\text {w.p. } P_\ell (i,t,\rho _{\bar{\ell }\ell })}, \text { if } x_i(t)=\text {R}_{\bar{\ell }},\\ \widetilde{\text {E}}_\ell , &{} \text {w.p. } P_\ell (i,t,\rho _{\ell \ell }), \text { if } x_i(t)=\text {R},\\ \end{array}\right. \end{aligned}$$

for $$\ell ,\bar{\ell }\in \{1,2\}$$.

### Network dynamics

To model the temporal evolution of the network of contacts ${\mathcal {G}}(t)=({\mathcal {N}} , {\mathcal {E}}(t))$, we adopt a discrete-time ADN (Perra et al. 2012). In this paradigm, each agent is associated with an activity potential $a_i$, which captures the individual’s social activity and tendency to initiate interactions with others within a single time step. The activity potential of individual *i* is a realization of a random variable from a distribution $f(a_i)$, where the activities are bounded by the inverse of the time step ($a_i\le \Delta ^{-1}$) to ensure that $a_i\Delta$ is a probability.

At each time instant *t*, each individual $i\in {\mathcal {N}}$ activates w.p. equal to $a_i\Delta$, independent of others. Each active individual will establish *m* undirected connections with others, generating the edge set ${\mathcal {E}}(t)$. The overall network dynamics can be organized into five main steps, which begin at $t=1$:

(i) The edge set is initialized as the empty set $\mathcal E(t)=\emptyset$;
(ii) Each individual $i\in {\mathcal {N}}$ becomes active w.p. equal to $a_i\Delta$, independent of others;
(iii) Each active individual $i\in {\mathcal {N}}$ selects *m* other individuals uniformly at random among the other individuals and establishes an undirected link with each of them, thereby forming the edge set;
(iv) Each individual $i\in {\mathcal {N}}$ updates its state $x_i(t)$ according to the transition mechanisms described in the “Node dynamics” sub-section and illustrated in Fig. 1; and
(v) The time step is updated to $t+1$.

All the parameters of the model are summarized in Table 1.

**Table 1:** Notation used in the paper

Notation	Meaning
*n*	Number of individuals
${\mathcal {N}}=\{1,\ldots ,n\}$	Population set
*t*	Discrete time variable
$\Delta$	Time step
${\mathcal {G}}(t)$	Time varying graph denoting the network of contacts
${\mathcal {E}}(t)$	Node set (interaction links) at time *t*
$x_i(t)$	State of individual *i* at time *t*
${\mathcal {X}}$	Discrete set of health states
$\text {S}$	Susceptible to both strains
$\text {E}_1$	Exposed to strain 1
$\text {E}_2$	Exposed to strain 2
$\text {I}_1$	Infectious with strain 1
$\text {I}_2$	Infectious with strain 2
$\text {R}_1$	Recovered from strain 1
$\text {R}_2$	Recovered from strain 2
$\widetilde{\text {E}}_1$	Exposed to strain 1 after being recovered from an infection
$\widetilde{\text {E}}_2$	Exposed to strain 2 after being recovered from an infection
$\widetilde{\text {I}}_1$	Infectious with strain 1 after being recovered from an infection
$\widetilde{\text {I}}_2$	Infectious with strain 2 after being recovered from an infection
$\text {R}$	Recovered from both strains
$\ell$	Index to denote a particular strain
$\lambda _\ell$	Per-contact infection probability of strain $\ell$
$\sigma _\ell$	Latency to become infectious of strain $\ell$
$\mu \ell$	Recovery rate for strain $\ell$
$\rho _{\ell \ell }$	Strain-specific re-infection probability for strain $\ell$
$\rho _{\ell \bar{\ell }}$	Cross-strain re-infection probability for strain $\bar{\ell }$
*m*	Average number of contacts per individual
$a_i$	Activity potential of individual *i*
$f(\cdot )$	Probability distribution of the activity potentials
$\langle {a}\rangle$	First order moment of the probability density function $f(\cdot )$
$\langle {a^2}\rangle$	Second order moment of the probability density function $f(\cdot )$
*T*	Time period of the control strategy
*D*	Duration of the home-isolation period
*p*	Fractions of home-isolated individuals in the control strategy

### Example

We illustrate our framework on a case study, with parameters inspired by COVID-19, to illustrate the repertoire of behaviors that our model can capture and reproduce. We consider a population of $N=10{,}000$ individuals and a time step equal to $\Delta =0.5$ day. Following (Behring et al. 2021; Parino et al. 2021), the transition probability from exposed to infectious and from infectious to recovered are set for both strains to $\sigma _{\ell }=0.5$ day$^{-1}$ and $\mu _{\ell }=0.2$ day$^{-1}$, respectively. To improve readability of the graphical presentation, we set the re-infection probabilities to $\rho _{\ell \bar{\ell }}=0.1$, for all $\ell ,\bar{\ell }\in \{1,2\}$, which is equivalent to a $90\%$ reduction of the probability to be infected due to natural immunity. Regarding the network dynamics, the value of the activity potential of each individual is drawn from a re-scaled power-law distribution $f(a)=\eta \,a^{-y}$ with exponent $y=2.1$, a cut-off $\epsilon =10^{-3}$, and re-scaling constant $\eta =10$. The number of connections per active individual is set to $m = 20$, based on literature (Mossong et al. 2008; Parino et al. 2021). As the initial condition, we consider one individual infected for each strain.

**Fig. 2:**
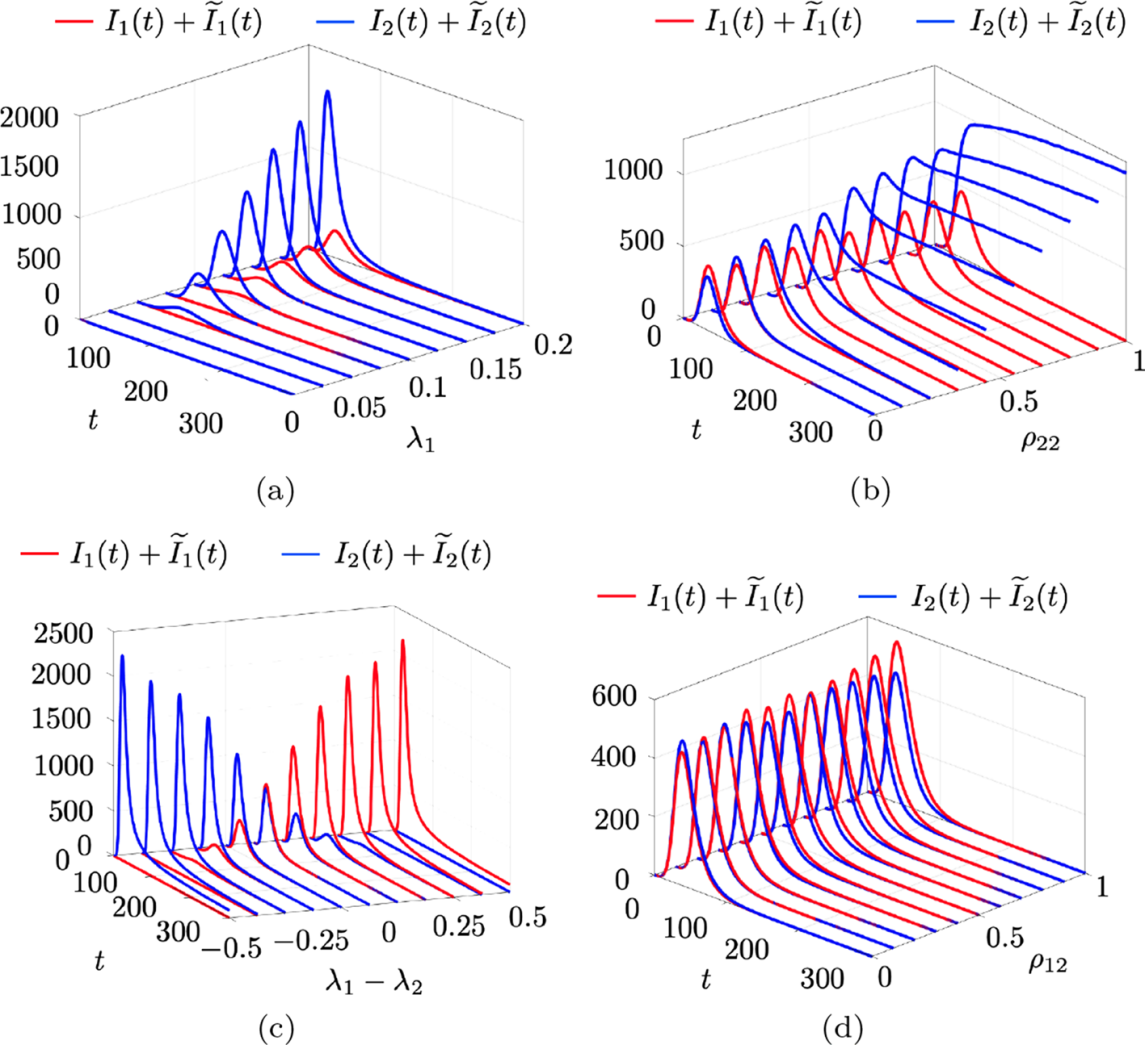
Illustrative example of the time evolution of the epidemic spreading process. Evolution of the epidemic in terms of the total infection counts for strain 1 ($I_1(t)+{\tilde{I}}_1(t)$) and 2 ($I_2(t)+{\tilde{I}}_2(t)$), averaged over 1000 independent Monte Carlo simulations for **a** different values of $\lambda _1$ with $\lambda _2 = 2\lambda _1$ being twice infectious than the first variant. Here $\lambda _1$ is varied from 0 to 0.2, thus representing cases where both variants are in the non-epidemic regime and transition to an epidemics as $\lambda _1$ increases. **b** Re-infection parameter of the second variant $\rho _{22}$ with $\rho _{21}=\rho _{22}$ and $\lambda _1=\lambda _2=0.2$. **c** $\lambda _1$ varies between 0 and 0.5, while $\lambda _2 = 0.5-\lambda _1$. **d** Number of re-infected individuals varying the cross-strain re-infection probability $\rho _{12}$ with $\lambda _1=\lambda _2=0.2$

Figure 2 illustrates the time evolution of the epidemic process for different values of the remaining parameters. In Fig. 2a, we vary the per-contact infection probability $\lambda _1$ from 0 to 0.2 while we consider the second strain to be two times more infectious than the first one, that is, $\lambda _2=2\lambda _1$. Predictably, the second variant dominates the infection count. In fact, once a critical threshold is trespassed, both strains yield an epidemic outbreak, but the second variant consistently leads the infection count at much higher figures than the first one.

The second set of simulations illustrates the role of the re-infection probability $\rho _{22}$ on the time evolution of the infection profile. In particular, in Fig. 2b, as the re-infection probability increases, the epidemic dynamics of the second variant exhibit a longer duration of the peak and a slower decay over time. Notably, for values of $\rho _{22}\ge 0.5$, the second strain tends to settle into an endemic regime that does not extinguish within the time interval of observation.

In Fig. 2c, we study how the interplay between the infection probabilities of the two strains affects the epidemic outcome. Specifically, we vary $\lambda _1$ from 0 to 0.5, and set $\lambda _2 = 0.5-\lambda _1$. All re-infection probabilities are set to $\rho _{\ell \bar{\ell }}=0.1$. Predictably, the results indicate that for $\lambda _1-\lambda _2<0$ the second variant is prevalent, for $\lambda _1-\lambda _2=0$ the two variants are equivalent, and for $\lambda _1-\lambda _2>0$ the first variant is, instead, prevalent. We also identify a transition from a disease-free steady-state value to an endemic state for each variant.

Finally, in Fig. 2d, we investigate the role of cross-immunity. Specifically, we vary the re-infection probability $\rho _{12}$ in [0, 1]. As expected, larger values of $\rho _{12}$ (low cross-immunity) lead to an increase in the number of infections from the second variant.

## Mean-field analysis

The example in Fig. 2 illustrates that our network epidemic model can exhibit three different types of emergent behaviors, namely,

(i) a *non-epidemic* regime, characterized by a quick convergence to a disease-free state, in which the infections monotonically decrease over time;
(ii) an *epidemic* regime, in which the number of infections grow initially, but, after reaching a peak, they vanish, eventually reaching a disease-free state; and
(iii) an *endemic* regime, where the disease persists over time and a disease-free state is never reached.

Here, we perform a theoretical analysis of the model to elucidate how model parameters determine the emerging behavior of the stochastic network system. Specifically, we derive two thresholds for the per-contact infection probability that characterize transition from the non-epidemic regime to the epidemic one, and from the epidemic regime to the endemic one, termed *epidemic threshold* and *endemic threshold*, respectively.

Following current practice in the study of ADNs (Perra et al. 2012; Liu et al. 2014; Rizzo et al. 2014, 2016; Zino et al. 2016; Lei et al. 2016; Pozzana et al. 2017; Ogura et al. 2019; Behring et al. 2021), we use a mean-field approach to approximate the time evolution of the total number of exposed and infected individuals using a set of nonlinear ordinary differential equations, in the limit $N\rightarrow \infty$ (Van Mieghem et al. 2009). In particular, we introduce the functions $I_\ell (\tau )$, $E_\ell (\tau )$, $R_\ell (\tau )$, as the continuous-time limit of the total number of individuals who are in the infected, exposed, and removed states of strain $\ell \in \{1,2\}$, when $\Delta \rightarrow 0$ (for clarity, we use $\tau$ for the continuous-time variable). Likewise, we use $S(\tau )$ and $R(\tau )$ to denote the total number of individuals in the susceptible and removed state, respectively.

Through a series of manipulations, detailed in “Appendix A”, we can establish that the dynamics of $I_\ell (\tau )$, $E_\ell (\tau )$ are governed by

6a $$\begin{aligned} \frac{\mathrm {d}I_\ell (\tau )}{\mathrm {d}\tau }&= - \mu _{\ell } I_\ell (\tau )+\sigma _{\ell }E_\ell (\tau )\,, \end{aligned}$$

6b $$\begin{aligned} \frac{\mathrm {d}E_\ell (\tau )}{\mathrm {d}\tau }&= -\sigma _{\ell }E_\ell (\tau )+\frac{m\lambda _{\ell }}{N}\left[ \Omega ^1_S(\tau )I_\ell (t)+S(\tau )\Omega ^1_{I_\ell }(\tau )\right] \\&\quad +\frac{m\lambda _{\ell }}{N}\left[ \Omega ^1_S(\tau ){\widetilde{I}}_\ell (\tau )+S(\tau )\Omega ^1_{{\widetilde{I}}_\ell }(\tau )\right] \\&\quad +\frac{m\rho _{\ell \ell }\lambda _{\ell }}{N}\left[ \Omega ^1_{R_\ell }(\tau )I_\ell (\tau )+R_\ell (\tau )\Omega ^1_{I_\ell }(\tau )\right] \\&\quad +\frac{m\rho _{\ell \ell }\lambda _{\ell }}{N}\left[ \Omega ^1_{R_\ell }(\tau ){\widetilde{I}}_\ell (\tau )+R_\ell (\tau )\Omega ^1_{{\widetilde{I}}_\ell }(\tau )\right] \,, \end{aligned}$$

for $\ell \in \{1,2\}$, respectively. Here, the function of time $\Omega ^d_{\bullet }(\tau )$ represents the *d*th order auxiliary variable that captures the *d*th moment of the activity of the individuals in the susceptible health state, up to the normalization constant *N*,

7a $$\begin{aligned} \Omega ^{d}_{S}(\tau )&:= \sum _{i\in {\mathcal {N}}:x_i(\tau )=\mathrm {S}}a_i^d\,, \end{aligned}$$

7b $$\begin{aligned} \Omega ^{d}_{I_\ell }(\tau )&:= \sum _{i\in {\mathcal {N}}:x_i(\tau )=\mathrm {I}_\ell }a_i^d\,, \end{aligned}$$

7c $$\begin{aligned} \Omega ^{d}_{{\widetilde{I}}_\ell }(\tau )&:= \sum _{i\in {\mathcal {N}}:x_i(\tau )=\widetilde{\mathrm {I}}_\ell }a_i^d\,, \end{aligned}$$

7d $$\begin{aligned} \Omega ^{d}_{R}(\tau )&:= \sum _{i\in {\mathcal {N}}:x_i(\tau )=\mathrm {R}}a_i^d\,.\quad \end{aligned}$$

The first and second summands in the right-hand side of equation (6a) denote the rate at which individuals leave and enter the infected state, respectively. Similarly, the first term in the right-hand side of equation (6b) identifies the rate at which individuals transition out from the exposed state to the infectious state. The second and third terms, instead, indicate the rate of transitions of susceptible individuals to the exposed state, after an interaction with individuals in $\mathrm {I}_\ell$ and $\widetilde{\mathrm {I}}_\ell$, respectively. The last two terms capture re-infections of individuals who have already recovered from the same strain, after an interaction with individuals infected with that strain or the other strain, respectively.

Analogously, the dynamics of the total number of individuals in the re-infected state ${\widetilde{I}}_\ell (\tau )$ and re-exposed state ${\widetilde{E}}_\ell (\tau )$ are governed by

8a $$\begin{aligned} \frac{\mathrm {d}{\widetilde{I}}_\ell (\tau )}{\mathrm {d}\tau }&=- \mu _{\ell } {\widetilde{I}}_\ell (\tau )+ \sigma _{\ell }{\widetilde{E}}_\ell (\tau ) \,, \end{aligned}$$

8b $$\begin{aligned} \frac{\mathrm {d}{\widetilde{E}}_\ell (\tau )}{\mathrm {d}\tau }&= -\sigma _{\ell }{\widetilde{E}}_\ell (\tau ) + \frac{m\rho _{\bar{\ell }{\ell }}\lambda _{\ell }}{N}\left[ \Omega ^1_{{R}_{\bar{\ell }}}(\tau )I_\ell (\tau )+ {R}_{\bar{\ell }}(\tau )\Omega ^1_{I_\ell }(\tau ) \right] \\&\quad +\frac{m\rho _{\bar{\ell }{\ell }}\lambda _{\ell }}{N}\left[ \Omega ^1_{{R}_{\bar{\ell }}}(\tau ){\widetilde{I}}_\ell (\tau )+ {R}_{\bar{\ell }}(\tau )\Omega ^1_{{\widetilde{I}}_\ell }(\tau ) \right] \\&\quad +\frac{m\rho _{\ell \ell }\lambda _{\ell }}{N}\left[ \Omega ^1_{R}(\tau )I_\ell (\tau )+R(\tau )\Omega ^1_{I_\ell }(\tau ) \right] \\&\quad +\frac{m\rho _{\ell \ell }\lambda _{\ell }}{N}\left[ \Omega ^1_{R}(\tau ){\widetilde{I}}_\ell (\tau )+R(\tau )\Omega ^1_{{\widetilde{I}}_\ell }(\tau ) \right] . \end{aligned}$$

The summands on the right-hand side of equation (8a) represent individuals that leave and enter the re-infected state. The first term on the right-hand side of equation (8b) denotes the rate of individuals who leave the exposed state and become (re-)infected. The second and third terms denote the rate of individuals who have already recovered from strain $\bar{\ell }$, and become exposed to strain $\ell$ after an interaction with individuals in ${\mathrm {I}}_\ell$ and $\widetilde{\mathrm {I}}_\ell$, respectively. The fourth and fifth terms capture the rate at which individuals who have already recovered from both variants and become again exposed after an interaction with individuals in ${\mathrm {I}}_\ell$ and $\widetilde{\mathrm {I}}_\ell$, respectively.

Finally, the dynamics of the first-order auxiliary variable are

9a $$\begin{aligned} \frac{\mathrm {d}\Omega ^1_{I_\ell }(\tau )}{\mathrm {d}\tau }&=- \mu _{\ell } \Omega ^1_{I_\ell }(\tau )+ \sigma _{\ell }\Omega ^1_{E_\ell }(\tau ) , \end{aligned}$$

9b $$\begin{aligned} \frac{\mathrm {d}\Omega ^1_{{\widetilde{I}}_\ell }(\tau )}{\mathrm {d}\tau }&= - \mu _{\ell } \Omega ^1_{{\widetilde{I}}_\ell }(\tau )+\sigma _{\ell }\Omega ^1_{{\widetilde{E}}_\ell }(\tau ) , \end{aligned}$$

9c $$\begin{aligned} \frac{\mathrm {d}\Omega ^1_{E_\ell }(\tau )}{\mathrm {d}\tau }&= -\sigma _{\ell }\Omega ^1_{E_\ell }(\tau ) +\frac{m\lambda _{\ell }}{N}\left[ \Omega ^2_S(t)I_\ell (t)+\Omega ^1_{S}(t)\Omega ^1_{I_\ell }(t)\right] \\&\quad +\frac{m\lambda _{\ell }}{N}\left[ \Omega ^2_S(t){\widetilde{I}}_\ell (t)+\Omega ^1_{S}(t)\Omega ^1_{{\widetilde{I}}_\ell }(t)\right] \\&\quad +\frac{m\rho _{\ell \ell }\lambda _{\ell }}{N}\left[ \Omega ^2_{R_\ell }(t)I_\ell (t)+\Omega ^1_{R_\ell }(t)\Omega ^1_{I_\ell }(t)\right] \\&\quad +\frac{m\rho _{\ell \ell }\lambda _{\ell }}{N}\left[ \Omega ^2_{R_\ell }(t){\widetilde{I}}_\ell (t)+\Omega ^1_{R_\ell }(t)\Omega ^1_{{\widetilde{I}}_\ell }(t)\right] , \end{aligned}$$

9d $$\begin{aligned} \frac{\mathrm {d}\Omega ^1_{{\widetilde{E}}_\ell }(\tau )}{\mathrm {d}\tau }&= -\sigma _{\ell }\Omega ^1_{{\widetilde{E}}_\ell }(\tau ) + \frac{m\rho _{\bar{\ell }\ell }\lambda _{\ell }}{N}\left[ \Omega ^2_{{R}_{\bar{\ell }}}(\tau )I_\ell (\tau )+ \Omega ^1_{{R}_{\bar{\ell }}}(\tau )\Omega ^1_{I_\ell }(\tau ) \right] \\&\quad +\frac{m\rho _{\bar{\ell }\ell }\lambda _{\ell }}{N}\left[ \Omega ^2_{{R}_{\bar{\ell }}}(\tau ){\widetilde{I}}_\ell (\tau )+ \Omega ^1_{{R}_{\bar{\ell }}}(\tau )\Omega ^1_{{\widetilde{I}}_\ell }(\tau ) \right] \\&\quad \frac{m\rho _{\ell \ell }\lambda _{\ell }}{N}\left[ \Omega ^2_{R}(\tau )I_\ell (\tau )+\Omega ^1_{R(\tau )}\Omega ^1_{I_\ell }(\tau ) \right] \\&\quad +\frac{m\rho _{\ell \ell }\lambda _{\ell }}{N}\left[ \Omega ^2_{R}(\tau ){\widetilde{I}}_\ell (\tau )+\Omega ^1_{R}(\tau )\Omega ^1_{{\widetilde{I}}_\ell }(\tau ) \right] . \end{aligned}$$

The dynamics of the auxiliary variables depend recursively on high-order auxiliary variables making the derivation of global results cumbersome. However, a local stability analysis can be conducted to shed light on the three different regimes, namely, non-epidemic, epidemic, and endemic, as articulated in what follows.

### Epidemic threshold

We start by analyzing the parameter conditions under which the non-epidemic behavior is observed. To this aim, we study the stability of the disease-free equilibrium of the stochastic network system in which all individuals are susceptible, that is, $S=N$ and all other variables are zero. The results of our analysis are summarized in the following claim.

#### Theorem 1

In the limit of large-scale networks $N\rightarrow \infty$, the non-epidemic behavior occurs when

10 $$\begin{aligned} \frac{\lambda _{\ell }}{\mu _{\ell }} < \frac{1}{m\left( \langle {a}\rangle +\sqrt{\langle {a^2}\rangle }\right) }\,, \end{aligned}$$

for both $\ell \in \{1,2\}$, where

11a $$\begin{aligned} \langle {a}\rangle :=\int _0^\infty af(a)\,\mathrm {d}a\,, \end{aligned}$$

11b $$\begin{aligned} \langle {a^2}\rangle :=\int _0^\infty a^2f(a)\,\mathrm {d}a\,, \end{aligned}$$

are the first- and second-order moments of the probability density function of the activity potentials.

#### Proof

By linearizing equations (6), (9a), and (9c) around the disease-free equilibrium $S=N$, we obtain

12a $$\begin{aligned} \frac{\mathrm {d}{I_\ell (\tau )}}{\mathrm {d}\tau }&= -\mu _{\ell }I_\ell (\tau ) +\sigma _{\ell }E_\ell (\tau ), \end{aligned}$$

12b $$\begin{aligned} \frac{\mathrm {d}{E_\ell (\tau )}}{\mathrm {d}\tau }&= - \sigma _{\ell } E_\ell (\tau )+{m\lambda _{\ell }}\left[ \langle {a}\rangle I_\ell (\tau ) + \Omega ^1_{I_\ell }(\tau )\right] , \end{aligned}$$

12c $$\begin{aligned} \frac{\mathrm {d}{\Omega ^1_{I_\ell }}(\tau )}{\mathrm {d}\tau }&= -\mu _{\ell }\Omega ^1_{I_\ell }(\tau ) + \sigma _{\ell }\Omega ^1_{E_\ell }(\tau ), \end{aligned}$$

12d $$\begin{aligned} \frac{\mathrm {d}{\Omega ^1_{E_\ell }(\tau )}}{\mathrm {d}\tau }&= - \sigma _{\ell } \Omega ^1_{E_\ell }(\tau )+{m\lambda _{\ell }}\left[ \langle {a^2}\rangle I_\ell (\tau ) + \langle {a}\rangle \Omega ^1_{I_\ell }(\tau )\right] \,, \end{aligned}$$

for $$\ell =\{1,2\}$$.

The stability of the disease free-equilibrium is fully determined by the stability of the origin of equation set (12) (Rugh 1996), which is determined by the Jacobian

13 $$\begin{aligned} \begin{bmatrix} -\mu _1 &{}\sigma _1 &{} 0 &{}0&{}0&{}0&{}0&{}0\\ m\lambda _1 \langle {a}\rangle &{}-\sigma _1 &{}m\lambda _1 &{}0&{}0&{}0&{}0&{}0\\ 0&{} 0&{}-\mu _1 &{}\sigma _1&{}0&{}0&{}0&{}0 \\ m\lambda _1 \langle {a^2}\rangle &{}0 &{} m\langle {a}\rangle \lambda _1 &{}-\sigma _1&{}0&{}0&{}0&{}0\\ 0&{}0&{}0&{}0&{}-\mu _2 &{}\sigma _2 &{} 0 &{}0\\ 0&{}0&{}0&{}0&{}m\lambda _2 \langle {a}\rangle &{}-\sigma _2 &{}m\lambda _2 &{}0\\ 0&{}0&{}0&{}0&{}0&{} 0&{}-\mu _2 &{}\sigma _2 \\ 0&{}0&{}0&{}0&{}m\lambda _2 \langle {a^2}\rangle &{}0 &{} m\langle {a}\rangle \lambda _2 &{}-\sigma _2\\ \end{bmatrix}\,. \end{aligned}$$

This $8\times 8$ matrix has a block-diagonal structure, so that its eight eigenvalues can be obtained by computing the eigenvalues of each of the $4\times 4$ diagonal blocks. Moreover, the structure of each block allows for an explicit computation of its four eigenvalues. In fact, the four eigenvalues of each block$\Lambda _{1,2,3,4}^{\ell }$ are the solution of the following equation:

14 $$\begin{aligned} \left[ (\mu _\ell +\Lambda ^{\ell })(\sigma _\ell -\Lambda ^{\ell })\right] ^2+\sigma _\ell m\lambda _\ell \langle {a}\rangle (\mu _\ell +\Lambda ^{\ell })(\sigma _\ell -\Lambda ^{\ell })-m^2\sigma _\ell ^2\lambda _\ell ^2\langle {a^2}\rangle =0. \end{aligned}$$

The solution of such an equation can be computed in closed-form, as

15 $$\begin{aligned} \Lambda _{1,2,3,4}^{\ell } := -\frac{\mu _{\ell }+\sigma _\ell }{2} \mp \frac{1}{2}\sqrt{(\mu _{\ell }-\sigma _{\ell })^2+4\sigma _{\ell }m\lambda _{\ell }\left( \langle {a}\rangle \mp \sqrt{\langle {a^2}\rangle }\right) }, \end{aligned}$$

with $\ell =\{1,2\}$. An epidemic outbreak does not occur if all the eigenvalues have negative real part, yielding the following condition:

16 $$\begin{aligned} \frac{\lambda _{\ell }}{\mu _{\ell }} < \frac{1}{m\left( \langle {a}\rangle +\sqrt{\langle {a^2}\rangle }\right) }\,, \end{aligned}$$

for both $\ell \in \{1,2\}$, which completes the proof. $\square$

Such a condition corresponds to the well-known threshold of SIS, SIR, and SEIR models with a single variant (Perra et al. 2012; Liu et al. 2014; Behring et al. 2021). Thus, the stability of the disease-free equilibrium in the presence of two strains is governed by the strain $\ell$ with higher ratio $\lambda _{\ell }/\mu _\ell$, that is, the strain which, on average, is able to infect more individuals during the entire transmissibility period. In fact, each infection occurs with per-contact transmission probability equal to $\lambda _\ell$, and the average duration of the transmissibility period is equal to $1/\mu _\ell$. This observation is in agreement with prior research on deterministic compartmental models (Fudolig and Howard 2020).

### Endemic threshold

The simulations in Fig. 2 suggest that some combinations of parameters yield regimes where the infection dynamics does not spontaneously extinguish. These regimes, called endemic, are of particular interest for the epidemiological community, as they underline scenarios where the population is required to “live with the virus (New York Times 2022).” Here, we determine a threshold, labeled as endemic, for the occurrence of this phenomenon.

#### Theorem 2

In the limit of large-scale networks $N\rightarrow \infty$, the endemic regime occurs if and only if

17 $$\begin{aligned} \frac{\lambda _{\ell }}{\mu _{\ell }} > \frac{1}{m\rho _{\ell \ell }\left( \langle {a}\rangle +\sqrt{\langle {a^2}\rangle }\right) }\,, \end{aligned}$$

for at least one $\ell \in \{1,2\}$, where $\langle {a}\rangle$ and $\langle {a^2}\rangle$ are defined in equation (11).

#### Proof

The determination of the endemic threshold is equivalent to isolating the conditions under which the dynamics does not converge to a disease-free state. To this aim, we study the stability of the equilibrium $R=N$ for the stochastic network system. By linearizing equation set (6), along with equations (9b) and (9d) about $R=N$, we obtain

18a $$\begin{aligned} \frac{\mathrm {d}{{\widetilde{I}}_\ell (\tau )}}{\mathrm {d}\tau }&= -\mu _{\ell }{\widetilde{I}}_\ell (\tau ) + \sigma _{\ell }{\widetilde{E}}_\ell (\tau )\,, \end{aligned}$$

18b $$\begin{aligned} \frac{\mathrm {d}{{\widetilde{E}}_\ell (\tau )}}{\mathrm {d}\tau }&= - \sigma _{\ell } {\widetilde{E}}_\ell (\tau )+{m\rho _{\ell \ell }\lambda _{\ell }}\left[ \langle {a}\rangle {\widetilde{I}}_\ell (\tau ) + \Omega ^1_{{\widetilde{I}}_\ell }(\tau )\right] \,, \end{aligned}$$

18c $$\begin{aligned} \frac{\mathrm {d}{\Omega ^1_{{\widetilde{I}}_\ell }}(\tau )}{\mathrm {d}\tau }&= -\mu _{\ell }\Omega ^1_{{\widetilde{I}}_\ell }(\tau ) + \sigma _{\ell }\Omega ^1_{{\widetilde{E}}_\ell }(\tau )\,, \end{aligned}$$

18d $$\begin{aligned} \frac{\mathrm {d}{\Omega ^1_{{\widetilde{E}}_\ell }(\tau )}}{\mathrm {d}\tau }&= - \sigma _{\ell } \Omega ^1_{{\widetilde{E}}_\ell }(\tau )+{m\rho _{\ell \ell }\lambda _{\ell }}\left[ \langle {a^2}\rangle I_\ell (\tau ) + \langle {a}\rangle \Omega ^1_{{\widetilde{I}}_\ell }(\tau )\right] \,. \end{aligned}$$

Following a procedure similar to the one used in the proof of Theorem 1, we evaluate the Jacobian of the system of equations at $R=N$, and we establish conditions for which one of the eigenvalues has a positive real part so that the equilibrium is unstable. Hence, we establish that

19 $$\begin{aligned} \frac{\lambda _{\ell }}{\mu _{\ell }} > \frac{1}{m\rho _{\ell \ell }\left( \langle {a}\rangle +\sqrt{\langle {a^2}\rangle }\right) }\,, \end{aligned}$$

for at least one $\ell \in \{1,2\}$, which yields the claim. $\square$

#### Remark 1

Both proofs in Theorems 1 and 2 rely on the block-diagonal structure of a Jacobian matrix, which begets two decoupled four-dimensional eigenvalue problems. Should one consider a multi-strain model (Paré et al. 2021; Fudolig and Howard 2020), with more than two strains, results would be equivalent.

**Fig. 3:**
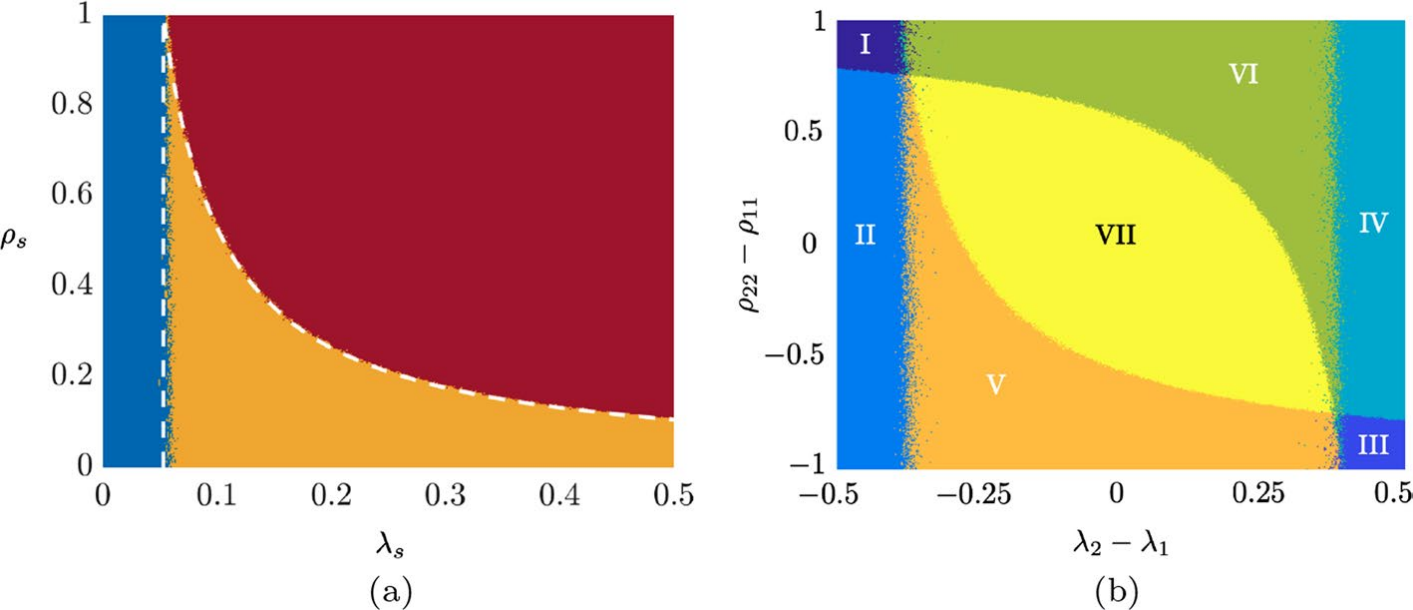
Two-dimensional diagram illustrating different types of behaviors of the stochastic network systems. In **a**, the two strains have equal infection and re-infection parameters. We vary the infection parameters $\lambda _1=\lambda _2=\lambda _s$ on the interval [0, 0.5], while the re-infection parameters $\rho _{11}=\rho _{22}=\rho _s$ are also varied on the interval [0, 1]. The blue region represents the non-epidemic regime, the orange the epidemic regime, and the red the endemic regime. Dashed lines indicate theoretical predictions. In **b**, we vary the infection and re-infection parameter values. Specifically, $\lambda _1$ and $\rho _{11}$ are varied on the interval [0, 0.5] and [0, 1], respectively, while we set $\lambda _2 = 0.5-\lambda _1$ and $\rho _{22} = 1-\rho _{11}$. Seven regions are highlighted, depending on the behavior of the two strains. In Region I, strain 1 is non-epidemic and strain 2 is epidemic; in Region II, strain 1 is non-epidemic and strain 2 is endemic; In Region III, strain 2 is non-epidemic and strain 1 is epidemic; In Region IV, strain 2 is non-epidemic and strain 1 is endemic; in Region V, strain 1 is epidemic and strain 2 is endemic; in Region VI, strain 2 is epidemic and strain 1 is endemic; in Region VII, both strains are endemic

We assess the validity of the epidemic thresholds in Theorems 1 and 2 through a series of simulations, in which we seek to map the parameter space into alternative behaviors of the stochastic network system. In particular, we create two-dimensional diagrams varying $\lambda _1=\lambda _2=\lambda _s$ and $\rho _{11}=\rho _{22}=\rho _s$ on the intervals [0, 0.5] and [0, 1], respectively. All other simulation parameters are the same as in the example in the “Example” sub-section. For each parameter combination, a total of 100 simulations were performed, each of 3600 time steps (see “Appendix B” for more details on the numerical simulations). Results are shown in Fig. 3a: the blue region indicates the non-epidemic regime where the disease monotonically vanishes in time, the yellow region identifies the epidemic regime in which an outbreak occurs but it is eventually eradicated, and the red region marks the endemic regime where the disease will persist over time. Each point is indicative of the average behavior observed over the 100 simulations.

The dashed white curves show theoretical predictions of the epidemic threshold. The vertical lines denote the epidemic threshold in (10) from Theorem 1, while the curves depict the endemic threshold in (17) from Theorem 2. Our results follow the intuition that highly infectious strains might enter the endemic region more easily, as they require lower values of the re-infection parameter $\rho _s$ for crossing the threshold.

Our theoretical claims from Theorems 1 and 2 clarify whether the stochastic network system will alternatively exhibit a quick eradication of the disease, an epidemic outbreak, or an endemic state. However, they do not allow for disentangling the infection count of each single strain. In particular, the two interacting strains can exhibit nontrivial behaviors, in which one of them is dominant or in which both strains coexist—two cases that are indistinguishable from our theoretical predictions. The analysis of these complex behaviors is nontrivial, and is still an open problem, even for models much simpler than ours (Liu et al. 2019; Paré et al. 2021; Doshi et al. 2021; Ye et al. 2022). Below, we conduct a numerical simulation campaign to provide insight into the complex spreading dynamics.

Through our simulations, we span different infection and re-infection parameter values: $\lambda _1$ and $\rho _{11}$ are varied in the intervals [0, 0.5] and [0, 1], respectively, while the parameters of the second strain are determined as $\lambda _2 = 0.5-\lambda _1$ and $\rho _{22} = 1-\rho _{11}$. In all the simulations, $\mu _1=\mu _2=0.2$. Note that, under these assumptions, Theorem 1 guarantees that the non-epidemic regime cannot occur, that is, at least one of the strains becomes epidemic (or endemic). To illustrate our findings, we color-code the behavior of the stochastic network system in a two-dimensional map, varying the infection parameters $\lambda _{1}-\lambda _{2}$ and re-infection parameters $\rho _{22}-\rho _{11}$ in the interval $[-0.5,0.5]$ and $[-1,1]$, respectively, as shown in Fig. 3b. For each combination, we perform 100 simulations over 3600 time steps (see “Appendix B” for more details on the numerical simulations).

Our numerical results highlight the non-trivial interplay of model parameters, which shape complex behaviors associated with seven different regions in Fig. 3b. Specifically, in Region I, strain 1 remains non-epidemic, while strain 2 yields an epidemic outbreak. In Region II, strain 1 remains non-epidemic, while strain 2 becomes endemic. Regions III and IV are characterized by a behavior symmetric to regions I and II, respectively (Region III: strain 2 remains non-endemic and strain 1 yields epidemic outbreak; Region V: strain 2 remains non-epidemic and strain 1 becomes endemic). In Region V, strain 1 exhibits an epidemic behavior, while strain 2 exhibits an endemic state, whereas the opposite occurs in Region VI. Finally, in Region VII, both strains exhibit an endemic state. Notably, regions I and III form the overall epidemic regime of the system, whereas the other regions pertain to the overall endemic regime. We should comment that two further regions may be possible, for other sets of parameters: a region in which the strains are non-epidemic, and a region in which both strains are epidemic—both regions are visible in Fig. 2a.

## Intermittent stay-at-home containment strategy

Our modeling framework can be used to inform containment policies. Here, we demonstrate its practical value by presenting the implementation of an intermittent stay-at-home strategy as a viable solution to mitigate the epidemic spread, while limiting the social and economic impact for the population. In particular, we analyze the effect of a stay-at-home containment strategy that involves randomly selected portions of the population to be home-isolated for limited time periods. We assume that home-isolated individuals are healthy during the isolation time and that they will remain healthy throughout the isolation period. Hence, in our simulations, we assign the “removed” state to these individuals, who temporarily do not contribute to the epidemic dynamics. More formally, we will randomly select a fraction $p\in [0,1]$ of the population to be home-isolated for a period of *D* consecutive time steps and we repeat this process every $T>D$ time steps.

Note that the total number of individuals who take part into the network dynamics are *N*(*t*), a number that changes in time according to a periodic switching law given by

20 $$\begin{aligned} N(t)&=\left\{ \begin{array}{ll} {(1-p)N,} &{} {kT\le t<kT+D,}\\ {N,} &{} {kT+D\le t <(k+1)T,} \end{array}\right. \end{aligned}$$

for $$k=\{0,1,\ldots \}$$.

By duplicating the mean-field analysis for this case, for each strain $\ell \in \{1,2\}$, we obtain a periodic, switched linear system of four coupled equations,

21a $$\begin{aligned} \frac{\mathrm {d}{I_\ell (\tau )}}{\mathrm {d}\tau }&= -\mu _{\ell }I_\ell (\tau ) + \sigma _{\ell }E_\ell (\tau )\,, \end{aligned}$$

21b $$\begin{aligned} \frac{\mathrm {d}{E_\ell (\tau )}}{\mathrm {d}\tau }&= - \sigma _{\ell } E_\ell (\tau )+{\omega _{\ell }(\tau )}\left[ \langle {a}\rangle I_\ell (\tau ) + \Omega ^1_{I_\ell }(\tau )\right] \,, \end{aligned}$$

21c $$\begin{aligned} \frac{\mathrm {d}{\Omega ^1_{I_\ell }}(\tau )}{\mathrm {d}\tau }&= -\mu _{\ell }\Omega ^1_{I_\ell }(\tau ) + \sigma _{\ell }\Omega ^1_{E_\ell }(\tau )\,, \end{aligned}$$

21d $$\begin{aligned} \frac{\mathrm {d}{\Omega ^1_{E_\ell }(\tau )}}{\mathrm {d}\tau }&= - \sigma _{\ell } \Omega ^1_{E_\ell }(\tau )+{\omega _{\ell }(\tau )}\left[ \langle {a^2}\rangle I_\ell (\tau ) + \langle {a}\rangle \Omega ^1_{I_\ell }(\tau )\right] \,, \end{aligned}$$

where $\omega _{\ell }(\tau )$ is a square wave,

22 $$\begin{aligned} \omega _{\ell }(\tau )&=\left\{ \begin{array}{ll} {m\lambda _{\ell }(1-p),} &{} {kT\Delta \le \tau<kT\Delta +D\Delta },\\ {m\lambda _{\ell },} &{} {kT\Delta +D\Delta \le \tau <(k+1)T\Delta } , \end{array}\right. \end{aligned}$$

for $$k=\{1,\ldots \}$$.

To study the stability of the periodic, switched linear system (21), we use Floquet theory (Rugh 1996). The transition matrix $\Phi (\tau ,\tau ')$ of any periodic linear system can be decomposed into

23 $$\begin{aligned} \Phi (\tau ,\tau ') = P(\tau )\exp \left( M(\tau -\tau ')\right) P^{-1}(\tau ')\,, \end{aligned}$$

where $\exp (\cdot )$ is the matrix exponential. The matrix function $P(\tau )$ is $T\Delta$-periodic, continuously differentiable, and invertible for all $\tau$, while *M* is a constant, possibly complex matrix that can be calculated from the monodromy matrix $\Phi (T\Delta ,0)$, as follows:

24 $$\begin{aligned} {M} := \frac{1}{T\Delta }\mathbf {\log } \left( \Phi (T\Delta ,0)\right) \,, \end{aligned}$$

with ${\log }(\cdot )$ being the matrix logarithm.

The Floquet decomposition can be used to transform the four-dimensional periodic system (22) into a time-invariant system, whose stability is dictated by the four eigenvalues of matrix *M*. For a switched system, the monodromy matrix takes the simple form of the product of matrix exponentials,

25 $$\begin{aligned} \Phi (T\Delta ,0):= \exp \big ((1-\delta ) T\Delta \,J_0\big )\exp (\delta T\Delta \,J_{p})\,, \end{aligned}$$

where $\delta =D/T$ is the duty cycle and

26 $$\begin{aligned} J_p:=\begin{bmatrix} -\mu _\ell &{} \sigma _\ell &{} 0 &{} 0\\ (1-p)m\lambda _\ell \langle {a}\rangle &{} -\sigma _\ell &{} (1-p)m\lambda _\ell &{} 0\\ 0 &{} 0 &{} -\mu _\ell &{} \sigma _\ell \\ (1-p)m\lambda _\ell \langle {a^2}\rangle &{} 0 &{} (1-p)m\lambda _\ell \langle {a}\rangle &{}- \sigma _\ell \end{bmatrix}\,. \end{aligned}$$

To investigate when an epidemic outbreaks occur for the switched, stochastic network systems, we examine the eigenvalues of *M*. By monitoring when the real part of at least one of these eigenvalues become positive, we pinpoint at the epidemic threshold. The same analysis can be performed around the equilibrium in which all the in individuals are in the $\mathrm {R}$ state to identify the endemic threshold, following the same steps as in the “Mean-field analysis” section.

**Fig. 4:**
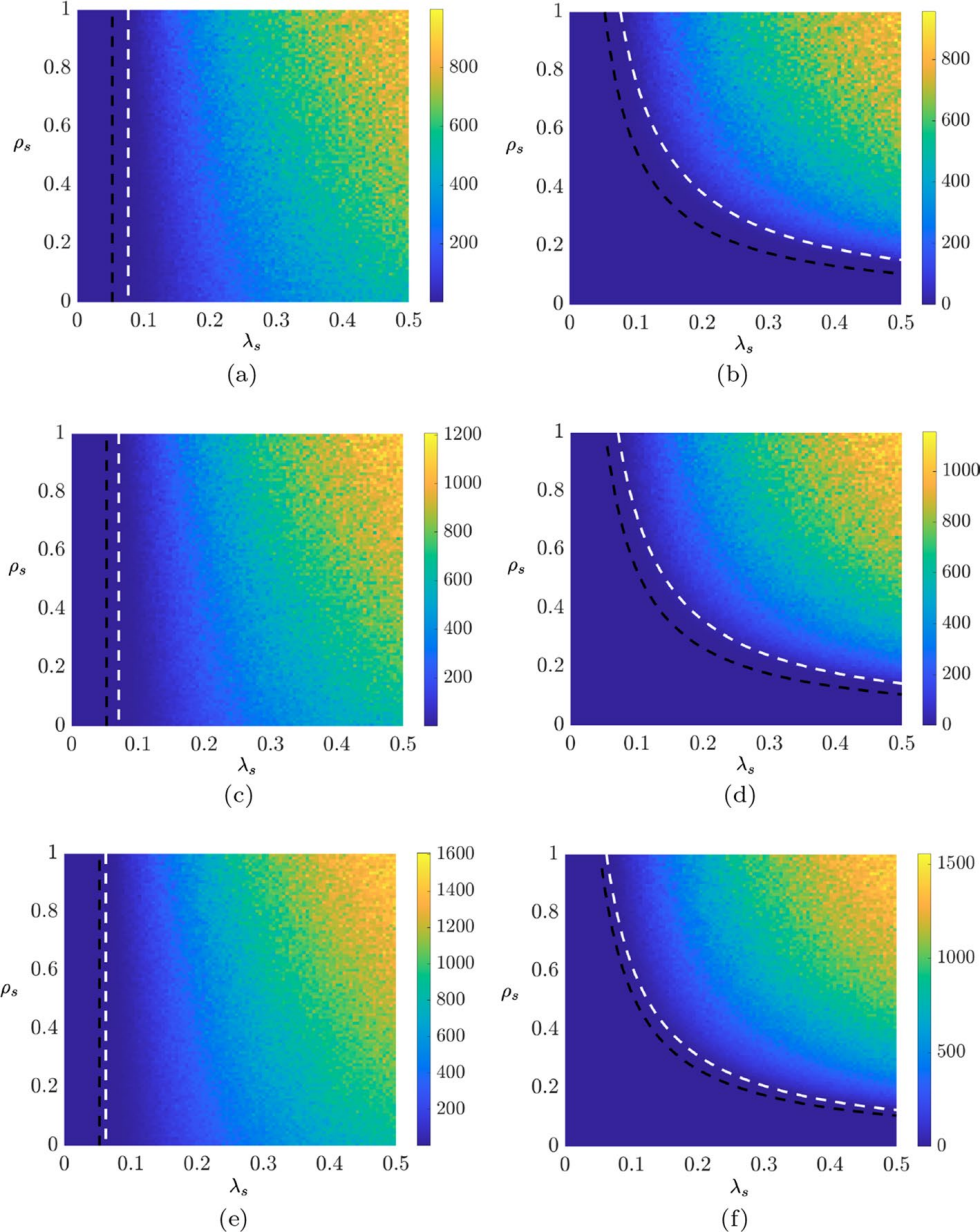
Two-dimensional diagrams illustrating the outcome of the intermittent stay-at-home containment strategy for three different values of the fraction of population: **a**, **b** $p=60\%$, **c**, **d** $p=50\%$, and **e**, **f** $p=30\%$. For each case, we report the peak count of infections (**a**, **c**, **e**) and the steady-state value (**b**, **d**, **f**), as determined from averaging the last 50 time steps. The white-dashed lines represent the stability thresholds computed from Floquet theory and the red dashed lines are stability threshold for $p=0\%$ (absence of the containment strategy, corresponding to Theorems 1 and 2)

The effect of the proposed intermittent stay-at-home containment strategy is illustrated in Fig. 4, through numerical simulations employing the same parameters as in the example in the “Example” sub-section. We perform 100 simulations for each parameter combination, each of 1000 time steps (see “Appendix B” for more details on the numerical simulations). We vary the fraction of individuals to be removed in the network *p*, and set the period to be 1 week ($T\Delta =7$), while the stay-at-home number of days is set to 5 days ($D\Delta =5$).

The dashed white curves represent the stability thresholds computed from the eigenvalues of *M* for both the epidemic and the endemic regimes. As the fraction of controlled nodes *p* increases, the region of stability of the disease-free equilibrium widens, while the one corresponding to the endemic regime shrinks. This can be observed by comparing the dashed white curves with the dashed red ones, which represent the stability thresholds in the absence of any containment strategy. In agreement with one’s intuition, both the peak count of infections and its steady-state value decrease for larger *p*. In fact, in the worst case scenario with $\lambda _1=\lambda _2=0.5$ and $\rho _{11}=\rho _{22}=1$, both values are reduced from more than 1500 cases per 10,000 inhabitants, to less than 1000 as *p* goes from 30 to 60%. To summarize, our results indicate that the presence of an intermittent stay-at-home containment strategy has a beneficial effect on the epidemic spreading. Not only can this strategy be used to mitigate new strains that might be more infectious than existing ones, but can it also be used to replace strict lock-down measures with long isolation periods.

## Conclusions

We developed and analyzed a two-strain epidemic model using the ADN paradigm. Building on state-of-the art models, we put forward a SEIR-based progression model that accounts for re-infections with the same strain or a different strain—scenarios that are presently unfolding during the COVID-19 pandemic as immunity is waning and new variants are emerging. The resulting model reveals rich dynamics through the stochastic network system that can experience different phenotypes, ranging from a disease-free equilibrium to epidemic outbreaks and endemic regimes in which the disease persists over time, through one of both its strains. Alongside computational insight, we establish closed-form expressions for the epidemic and endemic thresholds through a mean-field approach, which is valid in the thermodynamic limit of large networks. Predictably, the epidemic threshold is the same as the one corresponding to a classical SIS model over an ADN, when only the most infectious single strain is considered. In agreement with one’s intuition, the endemic threshold is inversely proportional to the strain-specific re-infection parameter.

We demonstrated the potential of the approach in the development of a stay-at-home containment strategy to mitigate the effects of the spread. Contrary to harsh lockdown measures that we have seen during the COVID-19 pandemic, this approach only requires that a small fraction of the population (selected uniformly at random) isolates for a period of time. After the isolation ends, individuals can return to normal activities and others will isolate in their place. We leverage Floquet theory to obtain the epidemic thresholds of such an intermittent strategy. We found that the region in the parameter space of the disease-free equilibrium grows with the fraction of individuals selected for isolation, thereby reducing the epidemic and endemic regions; a two-fold increase in the fraction of home-isolated inhabitants causes an equivalent drop in both the peak count of infections and its steady-state value.

Our proposed approach is not free of limitations and raises important questions to be addressed in future endeavors. First and foremost, the activity potential is assumed to be time-invariant, which may not fully capture the complexity of human behavior; for example, recent work from our group has demonstrated an extension of the ADN paradigm to account for memory effects through Hawkes’ processes (Zino et al. 2018) and for the inclusion of human behavior (Rizzo et al. 2014; Ye et al. 2021; Hota et al. 2022). Second, all individuals might not uniformly establish connections with others, rather, their interactions may be based on nodes’ properties (Pozzana et al. 2017), strong ties and dyadic relations (Sun et al. 2015; Nadini et al. 2020), or higher-order relations (Petri and Barrat 2018). Third, our containment strategy is open-loop, so it does not consider any feedback that could potentially enhance its mitigation, by anticipating outbreaks as reported in compartmental models (Della Rossa et al. 2020). Although there are several directions to be further explored, our results offer important insights into the dynamics and control of disease spreading processes with multiple strains over ADNs, an area which, to be best of our knowledge, was understudied till now.

## Data Availability

The simulation code is available in the online repository https://github.com/Dynamical-Systems-Laboratory/ADN-TwoStrains

## Appendix A: Mean-field dynamics

Mean-field theory (Van Mieghem et al. 2009) can be adapted to study the dynamics of the stochastic network system in (1)-(5). Consistent with Fig. 2, we utilize italic letters to quantify the number of individuals in each state of the progression model in Fig. 1. In particular we define *S*(*t*) and *R*(*t*) as the number of agents in the susceptible and recovered states a time *t*, respectively. Variables $E_1(t)$ and $E_2(t)$ and $R_1(t)$ and $R_2(t)$ count the number of exposed and recovered individuals from strain 1 and 2, respectively. Similarly, variables ${\widetilde{E}}_{1}(t)$, ${\widetilde{E}}_2(t)$, ${\widetilde{I}}_{1}(t)$, and ${\widetilde{I}}_{2}(t)$ count the agents in re-infected states, while *R*(*t*) is the total number of removed agents. Note that the total number of individuals *N* satisfies $N=S(t) + E_1(t)+E_2(t) + I_1(t)+I_2(t)+ R_1(t)+R_2(t)+{\widetilde{E}}_1(t)+{\widetilde{E}}_2(t)+ {\widetilde{I}}^{1}(t)+{\widetilde{I}}_2(t)+R(t)$.

Following (Perra et al. 2012), we consider a generic activity level *a* and we denote with a superscript *a* the number of individuals with activity level *a* in each state. For instance, $S^a(t)$ is the number of susceptible individual with activity *a* at time *t*. All the variables of the stochastic network system can be rewritten in a form analogous to the following one for the susceptible state and the related auxiliary variable:

27 $$\begin{aligned} S(t)= \int {S}^{a}(t)\mathrm {d}{a}\,,\qquad \Omega ^d_{S}(t)= \int a^d {S}^{a}(t)\mathrm {d}{a}\,, \end{aligned}$$

where, here and in what follows, integrals are all defined from 0 to $\infty$. Furthermore, for individual *i* with activity level *a*, we have that the expected value of (4) reads

28 $$\begin{aligned} {\mathbb {E}}[ {\mathcal {I}}_\ell (i,t)]=a\Delta m \int \frac{(I^{a'}_\ell (t)+{{\widetilde{I}}}^{a'}_\ell (t))}{N}\mathrm {d}{a'} +m \int \frac{a'\Delta (I^{a'}_\ell (t)+\widetilde{I}^{a'}_\ell (t))}{N}\mathrm {d}{a'}\,. \end{aligned}$$

Within the mean-field approach, we substitute $P_\ell (i,t,r)$ from (3) with its expected value, which is approximated by using (28), thereby obtaining

29 $$\begin{aligned} {\mathbb {E}}[P_\ell (i,t,r)]\approx 1-(1- r\lambda _{\ell }\lambda )^{\displaystyle\Delta m \left( a \int \frac{(I^{a'}_\ell (t)+{{\widetilde{I}}}^{a'}_\ell (t))}{N}\mathrm {d}{a'} + \int \frac{a' (I^{a'}_\ell (t)+\widetilde{I}^{a'}_\ell (t))}{N}\mathrm {d}{a'}\right) }\,. \end{aligned}$$

Such an expression is further simplified by considering a first-order McLaurin expansion in $\Delta$, which yields

30 $$\begin{aligned} P_\ell (i,t,r)\approx r\lambda _{\ell }\Delta m \left( a \int \frac{(I^{a'}_\ell (t)+{{\widetilde{I}}}^{a'}_\ell (t))}{N}\mathrm {d}{a'} + \int \frac{a' (I^{a'}_\ell (t)+\widetilde{I}^{a'}_\ell (t))}{N}\mathrm {d}{a'}\right) \,. \end{aligned}$$

Within a mean-field approach, for an infinitely large network $N\rightarrow \infty$, we can establish that the number of individuals exposed to strain $\ell \in \{1,2\}$ and belonging to the activity level *a* at a time $t +\Delta$ is approximated for small $\Delta$ by its expected value. From the dynamics described in the main article and recalling the progression illustrated in Fig. 1, we conclude that the change in the average number of exposed individuals to strain $\ell \in \{1,2\}$ belonging to the activity level *a* from time *t* to $t+\Delta$ is equal to the number of individuals who transition to $\mathrm {E}_\ell$ from $\mathrm {S}$ and $\mathrm {I}_\ell$ minus the number who transition from $\mathrm {E}_\ell$ to $\mathrm {I}_\ell$. Hence, we approximate $E^{a}_\ell (t+\Delta )$ as follows:

31 $$\begin{aligned} E^{a}_\ell (t+\Delta ) \approx&\,E^{a}_\ell (t) -E^{a}_\ell (t)\mathbb P\big [x_i(t+\Delta )= \text {I}_\ell \,|\,x_i(t+\Delta )= \text {E}_\ell , a_i=a\big ] \\&+ S^{a}_\ell (t){\mathbb {P}}\big [x_i(t+\Delta )= \text {E}_\ell \,|\,x_i(t+\Delta )= \text {S}, a_i=a\big ] \\&+R^{a}_\ell (t){\mathbb {P}}\big [x_i(t+\Delta )= \text {E}_\ell \,|\,x_i(t+\Delta )= \text {R}_\ell , a_i=a\big ] \end{aligned}$$

At this stage, from (1), we derive

32 $$\begin{aligned} {\mathbb {P}}\big [x_i(t+\Delta )= \text {I}_\ell \,|\,x_i(t+\Delta )= \text {E}_\ell , a_i=a\big ]=\sigma _\ell \Delta \,, \end{aligned}$$

while from (2), and using the approximation in (30) with $r=1$, we obtain

33 $$\begin{aligned} {\mathbb {P}}\big [x_i(t+\Delta )=&\text {E}_\ell \,|\,x_i(t+\Delta )= \text {S}, a_i=a\big ]\approx \\ \approx \,&a \, m\lambda _{\ell } \left( \int \frac{I^{a'}_\ell (t)}{N}\mathrm {d}{a'} \right) \Delta +a \, m\lambda _{\ell } \left( \int \frac{{\widetilde{I}}^{a'}_\ell (t)}{N}\mathrm {d}{a'} \right) \Delta \\&+m\lambda _{\ell } \left( \int \frac{a'I^{a'}_\ell (t)}{N}\mathrm {d}{a'} \right) \Delta +m\lambda _{\ell } \left( \int \frac{a'{\widetilde{I}}^{a'}_\ell (t)}{N}\mathrm {d}{a'} \right) \Delta \end{aligned}$$

Similar, from (5) and using the approximation in (30) with $r=\rho _{\ell \ell }$, we establish

34 $$\begin{aligned} {\mathbb {P}}\big [x_i&(t+\Delta )=\text {E}_\ell |x_i(t+\Delta )= \text {R}_\ell , a_i=a\big ]\approx \\ \approx \,&\rho _{\ell \ell }a \, m\lambda _{\ell } \left( \int \frac{I^{a'}_\ell (t)}{N}\mathrm {d}{a'} \right) \Delta +\rho _{\ell \ell }a \, m\lambda _{\ell } \left( \int \frac{{\widetilde{I}}^{a'}_\ell (t)}{N}\mathrm {d}{a'} \right) \Delta \\&+\rho _{\ell \ell }m\lambda _{\ell } \left( \int \frac{a'I^{a'}_\ell (t)}{N}\mathrm {d}{a'} \right) \Delta +\rho _{\ell \ell }m\lambda _{\ell } \left( \int \frac{a'{\widetilde{I}}^{a'}_\ell (t)}{N}\mathrm {d}{a'} \right) \Delta \end{aligned}$$

Finally, by replacing (32)–(34) into (31), we obtain the following approximation:

35 $$\begin{aligned} E^{a}_\ell (t+\Delta )\, {\approx }&\, E^{a}_\ell (t) - \sigma _{\ell } E^{a}_\ell (t)\Delta \\&+ S^a(t) \, a \, m\lambda _{\ell } \left( \int \frac{I^{a'}_\ell (t)}{N}\mathrm {d}{a'} \right) \Delta \\&+S^a(t)\, m\lambda _{\ell } \left( \int \frac{a'I^{a'}_\ell (t)}{N}\mathrm {d}{a'} \right) \Delta \\&+R^{a}_\ell (t) \, \rho _{\ell \ell }\, a \, m\lambda _{\ell } \left( \int \frac{I^{a'}_\ell (t)}{N}\mathrm {d}{a'} \right) \Delta \\&+R^{a}_\ell (t) \, \rho _{\ell \ell } \, m\lambda _{\ell } \left( \int \frac{a' I^{a'}_\ell (t)}{N}\mathrm {d}{a'} \right) \Delta \\&+ S^a(t) \, a \, m\lambda _{\ell } \left( \int \frac{{\widetilde{I}}^{a'}_\ell (t)}{N}\mathrm {d}{a'} \right) \Delta \\&+S^a(t)\, m\lambda _{\ell } \left( \int \frac{a'{\widetilde{I}}^{a'}_\ell (t)}{N}\mathrm {d}{a'} \right) \Delta \\&+R^{a}_\ell (t) \, \rho _{\ell \ell }\, a \, m\lambda _{\ell } \left( \int \frac{{\widetilde{I}}^{a'}_\ell (t)}{N}\mathrm {d}{a'} \right) \Delta \\&+R^{a}_\ell (t) \, \rho _{\ell \ell } \, m\lambda _{\ell } \left( \int \frac{a' {\widetilde{I}}^{a'}_\ell (t)}{N}\mathrm {d}{a'} \right) \Delta \,. \end{aligned}$$

The first summand on right-hand side of equation (35) corresponds to the number of individuals who are in the exposed state at time *t*. The second summand is the average number of individuals who transition from the exposed state to the infectious state. The third and fourth summmands correspond to the average number of individuals who transition from the susceptible state to the exposed one. Specifically, the third summand accounts for susceptible individuals with activity *a* who activate and interact with infectious individuals through the network of contacts ${\mathcal {G}}(t)=({\mathcal {N}} , {\mathcal {E}}(t))$; the fourth summand accounts instead for infected individuals who activate and interact with susceptible individuals with activity *a*. The fifth and sixth summands correspond to re-infection cases corresponding to the active and passive cases that transition in from the recovered state $R^{a}_\ell (t)$. Similarly, the last four summands correspond to incoming transitions due to interactions with re-infected individuals ${\widetilde{I}}^{a}_\ell (t)$. In addition, using equation (1), the number of infected individuals with strain $\ell$ and activity *a* at a time $t +\Delta$ is approximated by

36 $$\begin{aligned} I^{a}_\ell (t+\Delta )\,{\approx }\, I^{a}_\ell (t)+\sigma _{\ell }E^{a}_\ell (t)\Delta -\mu _{\ell }I^{a}_\ell (t)\Delta \,. \end{aligned}$$

Similar to equation (35), we approximate the number of re-exposed individuals at time $t+\Delta$ for small $\Delta$ with its expected value, computed following the same steps in (31)–(34), obtaining

37 $$\begin{aligned} {\widetilde{E}}^{a}_\ell (t+\Delta ) \,{\approx }&\, {\widetilde{E}}^{a}_\ell (t) - \sigma _{\ell }{\widetilde{E}}^{a}_\ell (t)\Delta \\&+ {R}^{a}_{\bar{\ell }}(t) \, a \, m\rho _{\ell \bar{\ell }}\lambda _{\ell } \left( \int \frac{I^{a'}_\ell (t)}{N}\mathrm {d}{a'} \right) \Delta \\&+{R}^{a}_{\bar{\ell }}(t)\, m\rho _{\ell \bar{\ell }}\lambda _{\ell } \left( \int \frac{a'I^{a'}_\ell (t)}{N}\mathrm {d}{a'} \right) \Delta \\&+ R^a(t) \, a \, m\rho _{\ell \ell }\lambda _{\ell } \left( \int \frac{{I}^{a'}_\ell (t)}{N}\mathrm {d}{a'} \right) \Delta \\&+R^a(t)\, m\rho _{\ell \ell }\lambda _{\ell } \left( \int \frac{a'{I}^{a'}_\ell (t)}{N}\mathrm {d}{a'} \right) \Delta \\&+ {R}^{a}_{\bar{\ell }}(t) \, a \, m\rho _{\ell \bar{\ell }}\lambda _{\ell } \left( \int \frac{{\widetilde{I}}^{a'}_\ell (t)}{N}\mathrm {d}{a'} \right) \Delta \\&+{R}^{a}_{\bar{\ell }}(t)\, m\rho _{\ell \bar{\ell }}\lambda _{\ell } \left( \int \frac{a'{\widetilde{I}}^{a'}_\ell (t)}{N}\mathrm {d}{a'} \right) \Delta \\&+ R^a(t) \, a \, m\rho _{\ell \ell }\lambda _{\ell } \left( \int \frac{{\widetilde{I}}^{a'}_\ell (t)}{N}\mathrm {d}{a'} \right) \Delta \\&+R^a(t)\, m\rho _{\ell \ell }\lambda _{\ell } \left( \int \frac{a'{\widetilde{I}}^{a'}_\ell (t)}{N}\mathrm {d}{a'} \right) \Delta \,. \end{aligned}$$

In addition, similar to (36), we approximate the number of re-infected individuals as

38 $$\begin{aligned} {\widetilde{I}}^a_\ell (t+\Delta )\,{\approx }\, {\widetilde{I}}^{a}_\ell (t)+\sigma _{\ell }{\widetilde{E}}^{a}_\ell (t)\Delta -\mu _{\ell }{\widetilde{I}}^{a}_\ell (t)\Delta \,. \end{aligned}$$

Taking the limit ${\Delta \rightarrow 0}$, equations (35)–(38) yield a set of ordinary differential equations for $E^{a}_\ell (\tau )$, $I^{a}_\ell (\tau )$, ${\tilde{E}}^{a}_\ell (\tau )$, and ${\tilde{I}}^{a}_\ell (\tau )$. For instance, from equation (36), by collecting all the terms in $\Delta$ on the right-hand-side, dividing by $\Delta$, and taking the limit, we find

39 $$\begin{aligned} \frac{\mathrm {d}I^{a}_\ell (\tau )}{\mathrm {d}\tau }=\sigma _{\ell }E^{a}_\ell (\tau )-\mu _{\ell }I^{a}_\ell (\tau )\,. \end{aligned}$$

A similar computation can be carried out for the other variables. Integrating across all the activity classes through (27) yields system of equations (6) and (8). To obtain the dynamics of the auxiliary variables, we multiply both sides of equations (35)–(38) by *a*, integrate across activity classes using (27), and take the limit $\Delta \rightarrow 0$, which yield (9).

## Appendix B: Numerical simulations

To create the two-dimensional diagrams in Fig. 3, we divided the parameter space of $\rho _s\in [0,1]$ and $\lambda _s\in [0,0.5]$ (or $\rho _{11}\in [0,1]$ and $\lambda _{1}\in [0,0.5]$) in a $400\times 400$ grid. For each parameter combination in the grid, we ran 100 independent simulations of the stochastic network system with one infected node per strain. The time window of each simulation was between 0 and 1800 days with a time step of $\Delta =0.5\,\mathrm {day}$ (3600 time steps). In all the simulations, we set $\sigma _\ell = 0.5\, \mathrm {day}^{-1}$ and $\mu _\ell = 2\,\mathrm {day}^{-1}$. To classify the behavior of each strain into the three possible regimes (non-epidemic, epidemic, and endemic), we follow the steps below.

- First, we average the solution across all trials.
- Second, we compute the peak and steady-state values of the number of infected with the strain. The steady-state is obtained as the time average of the last 50 time steps.
- Third, we classify the behavior of each strain as (i) non-epidemic, if the peak of the infection count is equal to one and the steady-state is below a tolerance $\varepsilon$ (that is, the infection count monotonically decays); (ii) epidemic, if the peak is above one and the steady-state below $\varepsilon$ (that is, the infection count has a peak before decaying toward the disease-free equilibrium); and iii) endemic if the peak is above one and the steady-state above $\varepsilon$. We heuristically selected $\varepsilon =0.1$ to be the steady-state tolerance.

To create the diagrams in Fig. 4, we utilized a coarser grid of $100\times 100$ and 500 days (1000 time steps). For each parameter combination in the grid, we ran 100 independent simulations of the stochastic network system with one infected node per strain. The diagrams report the peak count of infections and its steady-state value, considering both variants, averaged over the 100 independent simulations. The steady-state is obtained as the time average of the last 50 time steps.

## Abbreviations

ADN: Activity-driven network
ABM: Agent-based model
SIR: Susceptible–infected–removed
SIS: Susceptible–infected–susceptible
SEIR: Susceptible–exposed–infected–removed
w.p.: With probability
